# Salud sexual y reproductiva en mujeres adolescentes de programas de protección y reinserción social en Chile: tensiones entre paternalismo y autonomía corporal

**DOI:** 10.18294/sc.2025.5706

**Published:** 2025-12-09

**Authors:** Daniela González Aristegui, Ingrid Leal Fuentes, Carolina Carstens Riveros, Temístocles Molina González

**Affiliations:** 1 Autora de correspondencia. Magíster en Género y Cultura. Académica, Centro de Medicina Reproductiva y Desarrollo Integral del Adolescente, Facultad de Medicina, Universidad de Chile, Santiago, Chile. dgonzaleza@uchile.cl Universidad de Chile Centro de Medicina Reproductiva y Desarrollo Integral del Adolescente Facultad de Medicina Universidad de Chile Santiago Chile dgonzaleza@uchile.cl; 2 Magíster en Salud Pública. Académica, Centro de Medicina Reproductiva y Desarrollo Integral del Adolescente, Facultad de Medicina, Universidad de Chile, Santiago, Chile. igleal@uchile.cl Universidad de Chile Centro de Medicina Reproductiva y Desarrollo Integral del Adolescente Facultad de Medicina Universidad de Chile Santiago Chile igleal@uchile.cl; 3 Magíster en Antropología Aplicada en Salud y Desarrollo Comunitario. Profesional, Dirección de Igualdad de Género, Facultad de Medicina, Universidad de Chile, Santiago, Chile. ccarstens@uchile.cl Universidad de Chile Dirección de Igualdad de Género Facultad de Medicina Universidad de Chile Santiago Chile ccarstens@uchile.cl; 4 Magíster en Bioestadística. Académico, Centro de Medicina Reproductiva y Desarrollo Integral del Adolescente, Facultad de Medicina, Universidad de Chile, Santiago, Chile. tmolina@uchile.cl Universidad de Chile Centro de Medicina Reproductiva y Desarrollo Integral del Adolescente Facultad de Medicina Universidad de Chile Santiago Chile tmolina@uchile.cl

**Keywords:** Adolescentes, Poblaciones Vulnerables, Servicios de Salud Reproductiva, Chile, Adolescents, Vulnerable Populations, Reproductive Health Services, Chile

## Abstract

En una cultura adultocéntrica y adultista, las intervenciones estatales frente a vulneraciones de derechos de niñeces y adolescencias se sostienen en una mirada paternalista que limita su capacidad de ejercer autonomía a pesar de los tratados internacionales que la garantizan. En Chile, existen programas de protección y reinserción que intervienen conforme las necesidades que presenten a nivel familiar, comunitario e institucional, entre ellas de salud. Este estudio cualitativo exploratorio analizó 13 entrevistas a profesionales de la salud con experiencia en atención de adolescentes, realizadas en la Región Metropolitana entre abril de 2022 y abril de 2023, identificando la coexistencia de dos perspectivas de atención: una paternalista, centrada en el acompañamiento adulto, y otra orientada a favorecer la autonomía y el ejercicio de derechos. La perspectiva paternalista se basa en la percepción de riesgo y trauma, lo que puede restringir la autonomía en salud sexual y reproductiva. Se sugiere que las y los trabajadores de la salud integren una mirada interseccional para garantizar una atención pertinente y libre de juicios en salud sexual y reproductiva, incorporando el carácter relacional a la autonomía, especialmente en grupos vulnerados y prioritarios.

## Abstract (en)

In an adult-centered and adultist culture, state interventions addressing violations of the rights of children and adolescents are grounded in a paternalistic view that limits their ability to exercise autonomy, despite international treaties that guarantee it. In Chile, there are protection and reintegration programs that intervene according to the needs identified at the family, community, and institutional levels, including health-related needs. This exploratory qualitative study analyzed 13 interviews with health professionals experienced in adolescent care, conducted in the Metropolitan Region between April 2022 and April 2023, identifying the coexistence of two care perspectives: a paternalistic one, centered on adult accompaniment, and another oriented toward fostering autonomy and the exercise of rights. The paternalistic perspective is based on perceptions of risk and trauma, which may restrict autonomy in sexual and reproductive health. The study suggests that healthcare workers should integrate an intersectional approach to ensure relevant and nonjudgmental sexual and reproductive health care, incorporating the relational dimension of autonomy, particularly for vulnerable and priority groups.

## Introducción

Para favorecer el ejercicio de los derechos sexuales y reproductivos durante la adolescencia, es fundamental reconocer la autonomía que poseen las adolescencias en materia de sexualidad. Este reconocimiento debe darse por parte de la propia población adolescente en su calidad de usuaria del sistema de salud y sujeta de derechos, como también de quienes proveen atención. 

En una cultura adultocéntrica, el reconocimiento de la autonomía adolescente en materia de sexualidad dentro del ámbito de la salud se encuentra condicionado por las creencias de los equipos respecto de su capacidad de tomar decisiones sin la mediación de una persona adulta, así como por el temor a las posibles consecuencias médico-legales derivadas del desconocimiento de la normativa que respalda dicha autonomía^1^. De este modo, la influencia adulta incide directamente en las posibilidades de decisión de las y los adolescentes, lo que se intensifica en grupos prioritarios, quienes suelen ser concebidos principalmente como sujetos de protección, en tanto son representados por personas y/o instituciones en sus decisiones, reproduciendo enfoques paternalistas en la atención^2^.

La adolescencia, etapa comprendida entre los 10 y 19 años^3^, suele ser socialmente percibida como una etapa crítica debido a los múltiples cambios de carácter biopsicosocial que la caracterizan. Desde una mirada adultocéntrica, este período tiende a ser valorado de manera negativa, pues el proceso de construcción de identidades mediante la diferenciación respecto del entorno suele situar a las y los adolescentes en tensión con el mundo adulto^4^. 

En este período, si bien es esperable que comiencen relaciones sexoafectivas, ello no asegura que cuenten con condiciones materiales y simbólicas para ejercer sus derechos sexuales y reproductivos. La sexualidad continúa siendo un tema complejo de abordar para el mundo adulto por la ausencia intergeneracional de educación sexual integral^5^, la persistencia de mandatos de género que cuestionan la autonomía de las mujeres en materia de sexualidad^6^ y barreras de acceso a la salud sexual y reproductiva en el caso de adolescentes, sobre todo de grupos más vulnerados^7^^,^^8^^,^^9^. 

La autonomía progresiva es un principio fundamental reconocido en la Convención sobre los Derechos del Niño de las Naciones Unidas, que establece que adolescencias y niñeces son sujetos de derechos y deben participar en decisiones que les afectan, en función de su edad, madurez y desarrollo. El principio de autonomía progresiva se complementa con el interés superior, que establece que todas las decisiones y acciones que les afecten deben priorizar su bienestar y desarrollo integral^10^. La autonomía corporal está definida como la posibilidad y capacidad de decidir sobre el cuerpo sin coerción lo que implica poder decidir si se quiere tener relaciones sexuales, cuándo y con quién, el uso de métodos anticonceptivos y la asistencia a un centro de salud^6^. En la adolescencia, las posibilidades y capacidades de decidir sobre sus cuerpos se encuentran condicionadas por adultos: a nivel familiar, en las instituciones educativas y de salud, como también, a nivel legislativo. En este sentido es importante mencionar que, en caso de niñeces y adolescencias, la autonomía no implica autosuficiencia, en tanto posee un carácter relacional y colectivo que, para su ejercicio, requiere relaciones de interdependencia^11^. En grupos de adolescentes vulnerados en sus derechos, las posibilidades de ejercer autonomía disminuyen aún más por la tendencia adulta e institucional a observarles únicamente como sujetos de protección y pasivos frente a las decisiones que les competen^11^^,^^12^^,^^13^. 

Si bien el paternalismo moderno, como expresión adultocentrista, reconoce la existencia de derechos para las infancias y adolescencias^14^, tiende igualmente a un discurso protector que merma las posibilidades de protagonismo adolescente. Tanto familias como instituciones involucradas adoptan un rol tutelar que silencia y subordina^13^, estableciendo una relación asimétrica “*por su propio bien*”^11^.

En este sentido, la acción paternalista surge del genuino deseo de contribuir al bienestar de las personas, y en el caso de niñeces y adolescencias se justifica como algo inevitable que busca su beneficio^11^. Este paternalismo puede expresarse a nivel institucional, cuando el Estado toma decisiones por las personas o interviene sin previo consentimiento, lo que se denomina paternalismo jurídico^14^, accionar que tiende a observarse en mayor medida respecto de grupos vulnerados en sus derechos.

En Chile, las niñeces y adolescencias vulneradas suelen ser institucionalizadas a través de programas de protección y reinserción, que disponen de modalidad ambulatoria y residencial, implementados por servicios especializados que, en sus lineamientos técnicos, consideran la autonomía progresiva e interés superior. El servicio de protección considera una oferta programática dirigida a la restitución de derechos de niñeces y adolescencias vulneradas, y el servicio de reinserción cuenta con programas de integración social dirigidos a quienes han mantenido conductas al margen de la ley. Ambos servicios, además de acoger y dar respuesta al motivo de ingreso, deben favorecer la vinculación y el acceso a diferentes redes intersectoriales, entre ellas, la atención en salud. Para ello, como política intersectorial, desde el año 2017, se cuenta con el Sistema Intersectorial de Salud Integral (SISI), con énfasis en salud mental, que señala estrategias de trabajo conjunto para asegurar la priorización de esta población en particular^15^. En el año 2023, se publica una actualización de SISI que incluye líneas estratégicas específicas de atención en salud, dentro de las cuales se encuentra la salud sexual y reproductiva. 

Chile no cuenta con una ley de educación sexual integral (ESI), por lo que su incorporación en el currículo escolar es limitada^5^ y, con ello, la información a la que acceden las adolescencias para tomar decisiones en este ámbito resulta insuficiente, pese a que existen leyes que recientemente declaran la importancia de garantizar el ejercicio de sus derechos bajo el principio de autonomía progresiva^16^. Si bien en Chile ha disminuido el embarazo adolescente, posicionándose con las cifras más bajas de la región, y se ha incrementado el uso del preservativo junto a métodos anticonceptivos de larga duración^17^, se observa un incremento de otros indicadores de riesgo. Entre ellos, destacan el alza en las infecciones de transmisión sexual (ITS)^17^^,^^18^ y aumento de distintas expresiones de violencias de género en la población adolescente y joven en el contexto de relaciones interpersonales entre pares y sexoafectivas, especialmente en el espacio digital^17^^,^^19^. 

En este escenario, resulta crucial la labor de consejería que realizan los equipos de salud, tanto para la prevención como para el fortalecimiento de la toma de decisiones informadas en sexualidad. Esto es especialmente relevante en el caso de adolescentes que han vivido vulneraciones de derechos a lo largo de su trayectoria vital. Así, el SISI como política pública vigente, resulta clave al explicitar la necesidad de priorizar esta población en términos de acceso a servicios y atención^15^. Sin embargo, en una reciente revisión bibliográfica se ha observado que, pese al carácter prioritario que se señala respecto de este grupo de adolescentes, existen brechas que no garantizan el ejercicio de sus derechos, principalmente, por desconocimiento de la normativa y la mirada estereotipada que poseen ciertos profesionales del primer nivel de atención, lo que predispone a una atención diferenciada respecto de la población general^8^. 

El objetivo de este artículo es describir las perspectivas que poseen profesionales de salud del primer nivel de atención respecto de mujeres adolescentes que forman parte de programas de protección y reinserción, analizando la incidencia de esta mirada estereotipada en la atención que realizan. Se pone foco en la atención en salud sexual y reproductiva de mujeres, en tanto son quienes más acuden a estos servicios y quienes viven en mayor magnitud las consecuencias de no acceder a ellos oportunamente^7^^,^^8^^,^^9^. Se expondrá un análisis a partir de la experiencia directa, en diálogo con las nociones de paternalismo^14^ y autonomía corporal^6^^,^^20^ evidenciando las tensiones que existen. 

## Metodología

El enfoque metodológico adoptado fue de carácter cualitativo y exploratorio, en línea con el hecho de que no había suficiente evidencia nacional al respecto^21^, y con el objetivo de indagar en las perspectivas de profesionales del primer nivel respecto a la atención en salud sexual y reproductiva de adolescentes en programas de protección y reinserción social. Este enfoque resulta pertinente, pues permite comprender cómo las y los profesionales experimentan, interpretan y significan su práctica en torno a tensiones como la autonomía y el paternalismo, dando cuenta de discursos y prácticas situadas que difícilmente podrían captarse mediante técnicas cuantitativas. La elección de entrevistas cualitativas semiestructuradas se justificó por su capacidad de recuperar la voz de quienes trabajan en estas áreas, permitiendo explorar matices discursivos y profundizar en significados^22^. Se consideró que otras técnicas, como grupos focales u observación participante, no se ajustarían a los tiempos, condiciones éticas y al carácter sensible de la población y contexto de estudio.

Se utilizó un muestreo intencional^23^, con criterios de inclusión orientados a profesionales de salud del primer nivel de la Región Metropolitana, que tuvieran experiencia en la atención de adolescentes, integrantes de programas de protección y reinserción. Se buscó representar diversidad de género, disciplina y trayectoria laboral, garantizando además el criterio de saturación de la información, entendido como el momento en que la recolección de datos no aporta novedades relevantes para los objetivos del estudio^24^.

En total participaron 13 profesionales de la salud (11 mujeres y 2 hombres), cuyas edades fluctuaron entre los 27 y 41 años. La mayoría eran matronas, seguidas de trabajadoras sociales, terapeuta ocupacional y psicólogos y psicólogas. El promedio de experiencia laboral fue de nueve años, lo que aseguró un conocimiento situado del funcionamiento técnico-administrativo. El procedimiento de contacto inicial con las personas participantes fue realizado con el apoyo de una profesional del primer nivel encargada de reclutar a las personas que cumplían con el perfil solicitado, extendiéndoles la invitación a participar. Quienes aceptaban en esta primera instancia, eran contactados por integrantes del equipo investigador con experiencia previa en el primer nivel de atención y en el trabajo en red con programas de protección, quienes formalizaban la invitación. 

Las entrevistas cualitativas semiestructuradas se llevaron a cabo entre abril de 2022 y abril de 2023. Tuvieron una duración aproximada de 45 a 60 minutos y se realizaron en un espacio y horario acordado con cada participante en modalidad presencial y remota según preferencia. La pauta temática se derivó de los objetivos de la investigación y de la revisión de antecedentes teóricos en torno a adolescencia, salud sexual y reproductiva, derechos sexuales y reproductivos y sistemas de protección/reinserción, e incluyó dimensiones personales, contextuales e institucionales. Esta pauta fue validada con informantes claves del primer nivel de atención, servicios de salud y programas especializados en protección y reinserción, previa a la realización de la entrevista.

Las entrevistas fueron realizadas por las dos investigadoras principales formadas como matrona y trabajadora social, ambas con experiencia en atención, investigación y docencia en el área de salud sexual y reproductiva. Cada entrevista se registró mediante grabación de audio y notas de campo. Posteriormente, cada transcripción fue anonimizada mediante un código, eliminando toda información identificatoria y resguardando la confidencialidad de quienes participaron, en conformidad con las normas éticas vigentes. Para efectos de la presentación de resultados estos códigos incluirán profesión, género y edad para caracterizar el lugar de enunciación del relato. El proyecto contó con la aprobación del Comité de Ética de Investigación en Seres Humanos de la Facultad de Medicina de la Universidad de Chile (Nº 165-2021). 

El análisis de datos se realizó siguiendo los lineamientos del análisis de contenido cualitativo, combinando un enfoque inductivo y deductivo^25^. De manera deductiva, se construyeron categorías iniciales basadas en los objetivos de investigación y en referentes teóricos vinculados a la investigación. Paralelamente, se permitió la emergencia de categorías inductivas, de las que se desprendieron los temas del paternalismo y la autonomía, que emergieron del discurso de las personas participantes, posibilitando capturar hallazgos no previstos y dar voz a quienes participaron.

El procedimiento analítico incluyó las siguientes etapas: organización de datos, codificación inicial (realizada por el equipo de investigadoras), construcción y refinamiento de categorías, interpretación y contrastación con la bibliografía existente. Para garantizar la rigurosidad, se implementó validación inter-codificadores, triangulación entre investigadoras, descripción detallada de los contextos y participantes, así como comprobación de resultados con participantes y validación experta en un taller conformado por equipo especializado. El software MAXQDA fue utilizado como apoyo al proceso de análisis.

Se procuró cumplir con los criterios de credibilidad, transferibilidad, dependencia y confirmabilidad de la investigación cualitativa, resguardando la coherencia entre objetivos, técnicas y resultados^26^.

## Resultados

Para caracterizar la mirada que poseen las y los profesionales de la salud respecto del grupo adolescentes que forman parte de programas de protección especializada y reinserción social, con relación a la población general de adolescentes y permitir analizar la incidencia de esta visión preconcebida en la atención que realizan, se identificó como categoría teórica: *ideas preconcebidas sobre adolescentes que forman parte de programas de protección especializada y reinserción social*. A partir de este análisis, se identificaron dos categorías emergentes que coexisten en el quehacer profesional: la *perspectiva paternalista de la atención* y la *perspectiva de la atención centrada en la autonomía y ejercicio de derechos* (Tabla 1). 

**Tabla 1:** Categorías teóricas, categorías emergentes y dimensiones. Región Metropolitana, Chile, 2022-2023.

Categorías	Dimensiones
Categoría teórica	
Ideas preconcebidas sobre adolescentes que forman parte de programas de protección especializada y reinserción	Trayectorias de vida predeterminadas Falta de adherencia en salud Sexualidad desde el trauma
Categorías emergentes	
Perspectiva paternalista de la atención	Necesidad de acompañamiento adulto para asegurar la adherencia a las atenciones Necesidad del acompañamiento adulto por requerimientos administrativos Necesidad de acompañamiento adulto para decidir sobre un método anticonceptivo.
Perspectiva de la atención centrada en la autonomía y ejercicio de derechos	Respetar un espacio de atención confidencial Generar un espacio que favorezca el vínculo Evitar prejuicios asociados a programas de protección y reinserción social Fortalecer su proyecto de vida

Fuente: Elaboración propia.

### Ideas preconcebidas sobre adolescentes que forman parte de programas de protección especializada y reinserción social

En los relatos de profesionales de la salud se identifican ideas que instan a realizar atenciones diferenciadas a estas adolescentes respecto de la población general, atribuible al imaginario *a priori* respecto de lo que implica recibir una intervención en materias de protección y reinserción social. 

En este sentido, tienden a realizar generalizaciones respecto de las vivencias, sin contar necesariamente con los antecedentes, lo que queda de manifiesto en las observaciones realizadas respecto de las familias, asumiendo que son negligentes y, por ello, ausentes en la atención.

“*Es distinto a un chico que a una chica* […] *que no tenga situaciones de vulneración porque, en el fondo, ellos todavía, la mayoría tienen a sus papás bien encima, pero cuando sabes que hay negligencias en las familias, es súper complejo en realidad*”. (Psicóloga, 37 años)

Así mismo, suponen que por la historia de vida que poseen y que las y los determina, a diferencia de la población general de adolescentes, son otras las razones por las cuales eligen una pareja:

“*También pasa por el tema de ausencia de proyectos po’. No hay proyectos claros, no hay buenos rendimientos en el colegio, eh… no hay mucho acompañamiento a veces de nadie, entonces, cuando encuentran pareja, como que se aferran con uñas y dientes a las parejas que, habitualmente, son parejas mayores*”. (Matrona, 35 años)

Estas generalizaciones expresan un genuino interés profesional por subsanar esa carencia asumida, basándose en riesgos que suponen o creen que las adolescentes podrían enfrentar, las cuales se sumarían a las complejidades propias de la adolescencia.

“*Este grupo de niños, niñas, adolescentes y jóvenes ya ha pasado por vulneraciones, que por sí la niñez y la adolescencia tiene factores de riesgo asociados por el tema de la edad* […] *entonces sí po, hago más diferencia con estos jóvenes, porque sé que están con otro tipo de riesgos psicosociales, otro tipo de inequidades*...”. (Trabajador social, 31 años)

Esta motivación de proteger *a priori* se basa entonces en un aparente perfil que existiría en relación con este grupo y que orientaría la conducta a seguir como profesionales:

“*Las que vienen de estos programas y han, no sé, han tenido situación de calle, han pasado no sé, por distintos centros* […] *por distintas familias* […] *entonces a veces también están más vulnerables también en sus derechos, han sufrido violencia sexual…*”. (Matrona, 38 años)

Esta mirada centrada en la carencia sitúa a aquellos y aquellas profesionales que manifiestan mayor motivación, como responsables de contribuir, sin considerar la posibilidad de intervenir de manera conjunta con los programas de protección/reinserción, asumiendo que las adolescentes no cuentan con otros recursos para tomar decisiones en salud:

*“…es como también empezar a entregarles como a darles herramientas si finalmente hay que pensar que esos chicos van a salir a los 18 años de ahí* [en caso de adolescentes que viven en residencias de protección] *y que, ¿qué herramientas van a tener?*”. (Matrona, 42 años)

Respecto de las múltiples necesidades que van identificando y que justifican, a su criterio, la atención diferenciada, se observa cierta frustración en el hecho de que su accionar es acotado y no contribuye a detener las vulneraciones que puedan estar experimentando o la integración social que requieren, desconociendo la intervención que reciben en el ámbito de protección/reinserción, asumiendo que las materias de salud se realizan exclusivamente del sector salud y no de manera intersectorial.

“…*cuando yo logro, por ejemplo, darle prioridad a una adolescente vulnerable en colocarle un implante porque tiene mucho riesgo, eso es la punta del iceberg… yo necesito que un equipo entero entrara a intervenir conmigo a este chico*”. (Matrona, 35 años)

A partir de la misma predisposición, asumen y naturalizan la falta de adherencia al sistema de salud, dada la distancia que tendría esta población con la red y, por ende, la imposibilidad de realizar una labor preventiva como profesionales.

“…*es sabido de que toda la población ya viene siendo vulnerada en sus derechos y, por ende, también se da esta conducta de que no vienen a los controles, se alejan de este tipo de instituciones y, por lo general, uno sabe de ellos porque vienen al turno que es urgencia, pero no tiene esta lógica más preventiva*”. (Psicólogo, 32 años)

Pareciera ser que, a partir de un caso que les correspondió conocer de forma directa y que se comportó de cierta forma, construyen una percepción generalizada respecto de la relación de este grupo de adolescentes con el sector salud:

“*Tampoco a ellos se les hace la idea de ir al CESFAM* [centro de salud familiar] […] *ha sido muy difícil, porque no quieren, porque no les hace sentido, porque no lo necesitan. Entonces ese ha sido como un desafío*”. (Psicóloga, 32 años)

Así mismo, se asume la prevalencia de conductas de riesgo por sobre las conductas protectoras y, en caso de existir adherencia, se considera un hecho excepcional y atribuible al trabajo profesional:

“*Tengo una gestante en CIP* [Centro de Internación Provisoria] [...] *pensé que se iba a perder, y ¡no! ¡no se perdió!, viene a los controles puntuales, ha sido como súper adherente, entonces ya el lograr un poco de adherencia en los controles con ellos* […] *lograrlo más aún con ellos con todos estos factores de riesgo sociales que tienen es como súper enriquecedor*”. (Matrona, 27 años)

Respecto de la atención en salud sexual y reproductiva, se identifican ideas preconcebidas respecto de la sexualidad de este grupo específico de adolescentes, que predispone a las y los profesionales a suponer la existencia de traumas:

“...*yo creo que es una población que generalmente está como más traumatizada con todo lo que tiene que ver con la sexualidad, no solo de las relaciones sexuales, ¡todo!*”. (Psicóloga, 27 años)

Se establece una visión centrada en el riesgo, sin necesariamente tener antecedentes de la situación particular de la adolescente. Esta creencia estereotipada de la sexualidad de este grupo de adolescentes, eminentemente centrada en el riesgo, les hace evitar hablar de sexualidad en la atención de salud asumiendo que puede aumentar este trauma:

“…*a veces, me da un poquitito la impresión que sí pueden venir, quizás, un poquitito más revictimizados de repente* […] *a diferencia del otro grupo*”. (Psicóloga, 37 años)

El hecho de que formen parte de programas de protección y reinserción hace que el personal sanitario suponga que las adolescentes están expuestas a múltiples intervenciones, que mermarían su interés de abordar inquietudes respecto de sexualidad, en la atención en salud:

“…*el temor* [de hablar en el box de atención] *puede estar relacionado también al hecho de las tantas intervenciones que tienen los chicos, el temor a que se sepa mucho más de ellos* […] *yo creo que es la sobreintervención, yo creo que le tienen temor los chicos de poder expresar, decir lo que sienten, el tema de solicitar, por ejemplo, métodos anticonceptivos o de hacer preguntas con respecto a eso*”. (Matrona, 28 años)

Pero las ideas preconcebidas sobre las adolescentes no solo se limitan a generalizaciones en relación con las trayectorias de vida que las han llevado a programas de protección y reinserción, sino que también asumen un comportamiento futuro de sus decisiones sexuales y reproductivas, que incluso alcanzan sus planes de vida, que califican de ausentes:

“*Sí, habitualmente, tienen inicios sexuales más precoces, tienen búsqueda de afecto, siento yo, en la parte sexual. Eh… búsqueda de familias nuevas en las familias de las parejas, habitualmente. Búsqueda de integrarse a esas nuevas familias, a veces, con un embarazo… y… bastante ausencia de proyectos y de planes de vida*”. (Matrona, 35 años)

Respecto de las perspectivas que orientan el quehacer en salud y que predisponen a una intervención diferenciada con relación a la población general, se identifica mayoritariamente una mirada estereotipada respecto de las mujeres adolescentes que pertenecen a programas de protección y reinserción. Sin haber tenido necesariamente atenciones previas o acceso a sus antecedentes, como profesionales asumen una trayectoria de vida vulnerada, la pertenencia a familias negligentes y una vivencia traumatizada en la esfera de la sexualidad. Cabe preguntarse cómo incide esta visión preconcebida respecto a los procedimientos diferenciados de atención en salud que realizan con este grupo.

#### Perspectiva paternalista de la atención

Las ideas preconcebidas que poseen profesionales de la salud sobre este grupo de adolescentes se suman a las creencias en cuanto al acompañamiento adulto en las atenciones de salud sexual y reproductiva -pese a que la Ley 21418, que norma la regulación de la fertilidad en Chile, la indica solo respecto de la entrega de anticoncepción de emergencia- como también a los prejuicios en relación con la atención de adolescentes en general. De allí que los relatos de profesionales dan cuenta de que la participación de adultos se considera como una necesidad para el acceso, registro administrativo y atención intrabox, lo que es una barrera en aquellos casos donde se establece como requisito. 

En algunas situaciones, la inclusión de las personas adultas se justifica en la necesidad de asegurar la adherencia a las atenciones, la que no podría ser delegada al adolescente por características estereotípicas relativas al curso de vida.

“*Uno no puede delegar como la responsabilidad al adolescente de venir a sus controles, porque el adolescente es impulsivo, el adolescente no va a medir consecuencias. El adolescente es esperable que, hasta cierto punto, a lo mejor no le interese que tenga sus controles al día, que tenga sus controles dentales* [...] *sus controles médicos. Siempre es el adulto al cual se le exige que acompañe, en este caso, a adolescentes*…”. (Psicólogo, 32 años)

Respecto de esta población específica, señalan que es clave el acompañamiento de profesionales de los programas para aumentar la adherencia a las atenciones de salud, sobre todo en el caso de adolescentes de programas ambulatorios:

“…*cuando están con trabajador social o programa de acompañamiento vienen* [a la atención], *porque ahí le están reforzando todos los días, que tiene que venir, que tiene la próxima hora entonces ahí es como que más, mucho mayor su adherencia*”. (Matrona, 27 años)

La justificación de la necesidad del acompañamiento adulto se sustenta en que las plataformas de registro internas así lo requieren, exigencia administrativa que se desprende del modelo de salud familiar y que no se condice con las especificidades que presenta este grupo de adolescentes en cuanto a la posible ausencia de adultos que las acompañen en su acceso a la salud, lo que constituye una barrera:

“*En general el tema como es de salud familiar tiende a ser* [la incorporación de una persona adulta], *por lo menos los menores de 14… porque tienen que generar y vincularse con algún grupo de familia* [en plataforma de registro], *porque si pertenecemos a centros de salud familiar, entonces como que igual es raro no sé, tener un niño que esté solo y que no haya alguien que esté, que sea un tutor*”. (Matrona, 42 años)

En el caso de contar con otra figura adulta, como familias extensas o profesionales de los programas a los que pertenecen, queda de manifiesto una nebulosa respecto de los conductos administrativos, que no contemplan la compañía de adultos sin vínculos consanguíneos o tutorías legales:

“*Ahí me queda un poco la duda del tema de la inscripción* [...] *porque igual se per capitaliza* [procedimiento de evaluación socioeconómica al ingreso al centro de salud] *con la huella dactilar, pero está a cargo de alguien… entonces a lo mejor podría ser un alguien, por último, un tutor, con este contexto socioeconómico, o sea social, socio… psicosocial finalmente, a lo mejor de alguna manera ayudarlo así ingresar al sistema de salud*”. (Matrona, 42 años)

Junto a ello, tanto la familia, como profesionales de la salud y de otros programas justifican la necesidad de incidencia adulta en la decisión del inicio y uso de un método anticonceptivo. Desde salud, si bien existe la noción de que ello podría afectar el ejercicio de sus derechos, se valida arbitrariamente la necesidad de una decisión compartida entre adolescente y una persona adulta, naturalizando el hecho de que sean las personas adultas quienes planteen el motivo de consulta, proponiendo el uso de un método anticonceptivo bajo un criterio disciplinario y/o una decisión arbitraria. Lo anterior queda expresado en la siguiente situación intrabox recreada por una profesional dentro de la entrevista,

“[matrona le pregunta a la adolescente] *¿por qué viene?* [adolescente dice] *porque me trajeron, porque me obligaron, porque la tía dijo que tenía que venir… y a veces me dicen: es que la tía me dijo que tenía que ponerme un método anticonceptivo* […] *es que mi mamá dice que venga, si no, no me va a dejar salir, es que la tía dice que, si no tengo método, no me van a dar permiso, no sé, para ver a alguien*”. (Matrona, 38 años)

#### Perspectiva de la atención centrada en la autonomía y ejercicio de derechos

En las experiencias compartidas, junto con una perspectiva paternalista, convive una mirada centrada en la autonomía, que tiene en cuenta las particularidades de este grupo, para comprender las necesidades e intereses que presentan en salud. 

Como principal expresión de reconocimiento de la autonomía, señalan la importancia de respetar un espacio de atención confidencial. Esto lo fundamentan en la posibilidad de abordar aspectos relativos a su sexualidad que no sería posible con la presencia de algún adulto, ya sea familiar o profesional de los programas en los cuales participan:

“*Yo personalmente cuando veo a una adolescente mayor de 14, pido que pase sola al box, porque también entiendo que cuando viene con la mamá o con el adulto que están no van a conversar*”. (Matrona, 38 años)

Pese a lo anterior, sobre todo en el caso de los menores de 14 años -edad legal de consentimiento sexual en Chile- preocupa que si bien la normativa vigente solo indica acompañamiento adulto en el caso de anticoncepción de emergencia y la persona adulta que la adolescente determine, en la práctica, se hace extensiva a otras circunstancias:

“*Generalmente intento intencionar de que esté el adulto al principio para que se entere, y luego le solicito tener este espacio como más privado* […] *pero si hay algo que interfiera …y que vaya a causar daño en la vida del adolescente se le va a comunicar*”. (Matrona, 42 años)

“*Cuando son menores de 14, por una cosa legal, si van a iniciar método tiene que venir con una acompañante, pero les explico todo y siempre trato de tener esos resguardos*”. (Matrona, 38 años)

Otro aspecto que relevan es la preocupación de generar un espacio que favorezca el vínculo entre adolescente/profesional y la red de salud. Para ello, se destaca la importancia de evitar juicios respecto de las decisiones que toman las adolescentes en el contexto de una atención,

“…*yo intento con cada una de las pacientes que se sienta lo más cómodo posible y que la idea es que vuelva*”. (Matrona, 42 años)

Desde la experiencia que tienen como profesionales, este espacio seguro y libre de juicios es identificado por las adolescentes, impactando en la adherencia y continuidad de los cuidados,

“…*me empecé a dar cuenta que a los únicos controles que no faltaba era cuando eran conmigo, entonces como era una adolescente vulnerable, tenía un contexto social súper complejo, la empecé a citar, 2 semanas, ¡20 minutos! pero eran 20 minutos que ella conversaba, se iba y después volvía* [...] *no nos pasaba eso con todas las atenciones, entonces esas pequeñas cositas como que me fueron enseñando cómo ir trabajando*”. (Matrona, 27 años)

Para asegurar un espacio seguro y pertinente a sus necesidades, se explicita la relevancia de observar los distintos factores que inciden en su vivencia, evitando así ideas preconcebidas, exclusivamente asociadas a su vínculo con programas de protección y reinserción social,

“…*es diferente que una adolescente sea migrante, que sea, no sé indígena, entonces son otro tipo de situaciones que aumentan los riesgos, como desde una teoría más de la interseccionalidad cierto, entonces son capas, y van afectando más a la persona*”. (Trabajador social, 31 años)

Esto implica tener disposición de brindar una atención pertinente a sus necesidades y motivaciones, evitando que la consejería esté predeterminada por la vulneración de sus derechos y/o hayan cometido conductas al margen de la ley:

“…*como que no hago mucha diferencia* […], *o sea, más allá de la vulneración de derechos que pudo haber tenido en su vida, o no sé, si está judicializada por robo… no hago mucha diferencia en la consejería porque creo que no va asociado al programa que esté, sino que va como a su etapa del ciclo vital, entonces como que trato de hablar con todas algo como similar*”. (Matrona, 38 años)

Se enfatiza la necesidad de garantizar la atención prioritaria a este grupo de adolescentes, por ende, la diferenciación estaría en el acceso prioritario no en la atención, lo que implica que a nivel de equipos puedan considerar una mayor flexibilidad para disminuir la existencia de brechas.

“*No hay ninguna diferencia en la atención que uno le hace, ninguna* […] *la única diferencia podría ser que, si un chico derivado de tribunales o de alguna entidad reparatoria, requiere una atención más urgente, le demos una hora más rápido, podría ser*”. (Matrona, 35 años)

“…*adolescentes en situación de calle, por ejemplo, que no necesariamente van a tener un domicilio fijo en la comuna* […] *Igual se atienden*”. (Matrona, 42 años)

Considerar la especificidad de sus vivencias requiere que los equipos puedan ajustarse a situaciones que van más allá de la responsabilidad de agendar atenciones o asistir con puntualidad, en tanto inciden aspectos contextuales/estructurales que no dependen de ellas: vivir en calle, no contar con un adulto responsable consanguíneo, trasladarse continuamente de domicilio o que su situación migratoria no se encuentre regularizada:

“…*me ha pasado que han venido chiquillas solas o a veces acompañadas del mismo pololo* [novio] *que a veces tiene hasta la misma edad, entonces claro el acceso uno no puede negarlo, si no viene el adulto responsable después de los 14 también tenemos el resguardo de poder atenderlos igual*”. (Matrona, 42 años)

Consideran que la intervención, particularmente en el ámbito de la salud sexual y reproductiva, debe enfocarse en fortalecer la autonomía respecto de su proyecto de vida. Al igual que en la atención de la población general de adolescentes, conocer las expectativas e intereses en el contexto de la atención, brinda un espacio de oportunidad para un acompañamiento que visibilice los recursos que posee, al reforzar su proyecto de vida:

“*Lo que sí podemos propiciar es que termine sus estudios, que no se embarace. Incentivar algún proyecto de vida, como reforzar sus propias herramientas personales para que la maternidad o el quedarse en la casa no sea una opción, al menos, no en este momento*”. (Psicólogo, 30 años)

Como profesionales, enfatizan que las decisiones son parte de sus derechos como adolescentes, y el rol como profesionales es facilitar la información para que los ejerzan de manera informada y oportuna, libre de juicios:

“…*yo soy super empática porque le digo, no puedo elegir yo, no te puedo decir que usar, yo te puedo explicar un poco, pero mira y es que hoy día por una cosa de redes sociales, las adolescentes tienen mucho más acceso a la información, entonces vienen un poco más predispuestas y saben también un poco más*”. (Matrona, 38 años)

De las ideas preconcebidas que actúan como estereotipos respecto de este grupo de adolescentes, se desprenden dos perspectivas contrapuestas que conviven en la atención de adolescentes que pertenecen a programas de protección y reinserción social, es decir, una persona puede tener consideraciones paternalistas y, al mismo tiempo, centradas en la autonomía corporal. Estas ideas preconcebidas -generalización de sus vivencias y su grupo familiar, una visión de la sexualidad centrada únicamente en el trauma y la naturalización de su falta de adherencia en salud- determinan la visión que poseen en cuanto a la trayectoria de vida que han tenido, y también inciden al presumir la ocurrencia futura de otros eventos en el ámbito de la salud sexual y reproductiva. 

Lo anterior, sumado a una perspectiva paternalista de la atención adolescente en salud sexual y reproductiva que observa la necesidad de incorporación adulta, podría constituirse como barrera de acceso para este grupo de la población en particular, en tanto las adolescencias perciben la existencia de prejuicios por parte de profesionales. Si bien la convivencia de estas ideas con una mirada centrada en la autonomía corporal podría subsanar estos posibles obstáculos, en el ejercicio de derechos sexuales y reproductivos se requiere de una revisión crítica de ideas preconcebidas para evitar que se constituyan como barreras, como también a nivel de equipos para una consideración multidisciplinaria en la comprensión y abordaje, sobre todo de grupos que presentan mayores factores de exclusión y, por ello, requieren de prioridad. 

## Discusión

Desde el construccionismo social, se entiende por perspectiva la manera en que las personas interpretan y construyen la realidad a partir de su contexto social, cultural e histórico. Esto implica que la realidad se construye socialmente^27^, lo cual se expresa en el quehacer en salud en una predisposición, en tanto nuestra forma de ver el mundo incide en lo que consideramos como una verdad^28^. 

La perspectiva es entonces, una visión de la realidad que incide en la manera en que nos enfrentamos a diferentes situaciones. En el caso de la experiencia de profesionales de la salud, que se desempeñan en el ámbito de la salud sexual y reproductiva, se observan ideas preconcebidas respecto de las adolescencias que forman parte de programas de protección y/o reinserción, en las que coexisten dos perspectivas contrapuestas de la atención que brindan: una paternalista y una centrada en la autonomía y en los derechos. 

En las definiciones de paternalismo podemos encontrar dos variantes: la tradicional que consiste en la subordinación total de niñeces y adolescencias frente a la figura del adulto, y la moderna, que si bien les identifica como sujeto de derechos, no favorece las condiciones para la participación en su ejercicio, manifestando con ello, un discurso ambivalente^14^. En este caso, se considera un accionar paternalista, en tanto la persona adulta -profesional de la salud- interviene, a pesar de que la adolescente hubiera sido capaz de tomar una decisión propia que corresponda a sus intereses, siendo insuficiente el argumento de cautelar su bienestar^11^. Lo que hace entonces paternalista una actitud es “*volver al niño/a más pequeño o más débil de lo que realmente es*”^14^, pudiendo compensar las posibles desventajas que pueda presentar, acompañando el ejercicio de su autonomía desde un punto de vista relacional^11^. 

El paternalismo se considera una actitud adultocéntrica o adultista, en tanto prevalece la autoridad adulta respecto de la autoridad de niñeces, adolescencias y juventudes, lo que corresponde a la hegemonía de la sociedad, estructurada en torno a la adultez, perpetuando un mecanismo de desigualdades^12^. Este tipo de actitudes tienden a justificarse en la protección que se pretende brindar mediante la intervención adulta, instalándose la idea de vulnerabilidad *per se* en las infancias y adolescencias, restando su capacidad de agencia, entendida como la participación protagónica que les reconoce como actores-activos en la construcción de sus vidas y en su incidencia en el contexto en el que viven^29^. En este caso, el accionar de las y los profesionales de la salud, fundamentado en ideas preconcebidas, insta a asumir que existe negligencia familiar sin cotejar que efectivamente es así su situación, desde una mirada esencialista que les predispone a una atención desde la carencia. 

En este sentido, la existencia de normativas en salud sexual y reproductiva, que resguardan la autonomía de adolescentes, no necesariamente asegura su reconocimiento, en tanto las nociones estereotipadas de las personas adultas que les proveen atención -en este caso factores de riesgo a nivel familiar, falta de adherencia del sistema de salud y visión de una sexualidad traumática- caracterizan a las adolescencias como entes pasivos frente a las decisiones que les competen en este ámbito y carentes respecto de los recursos que poseen. Lo anterior, pese a existir, como marco general, la Convención sobre los Derechos del Niño y, en lo específico en Chile, la Ley 20418, con relación a la salud sexual y reproductiva, que cautela el acceso a atención y educación en materia de regulación de la fertilidad desde los 14 años en adelante^30^. Por lo tanto, la mera existencia de normativas no incide en el desmantelamiento de las relaciones jerárquicas de una cultura adultista^14^. 

Respecto de ello, un estudio sobre la práctica clínica de proveedores de salud en la atención adolescente señala que, los equipos tienden a desconocer aspectos legales que resguardan la confidencialidad en sexualidad de esta población, por los posibles conflictos que ello podría conllevar a nivel del quehacer institucional, generando barreras en el acceso a una atención oportuna^1^. Esto se condice con la relación conflictiva que se reconoce propia de sociedades adultocéntricas^12^^,^^13^, como también de un paternalismo jurídico en el que las instituciones toman decisiones por las personas sin un consentimiento previo, reduciendo su libertad y autonomía^14^. 

Con relación a los resultados de este estudio, la noción de paternalismo moderno y jurídico caracteriza las perspectivas que se expresaron en el quehacer en salud respecto de este grupo de adolescentes. Aun cuando los equipos reconocen la condición de las adolescencias como sujetos de derecho, pese a ello, existe una tendencia a prevalecer la mirada de los adultos que forman parte de sus vidas: familia de la adolescente, acompañante del programa de protección o reinserción social y/o profesional de salud que realiza la atención. Si bien la justificación de ese accionar paternalista es diversa y se presenta en términos positivos -reforzar adherencia, requerimiento administrativo, asesorar inicio de método anticonceptivo y/o entregar información- se relaciona con ideas preconcebidas respecto de este grupo en específico y no con aspectos propios de la atención. 

En los equipos profesionales existe claridad respecto de que las adolescencias son sujetos de derechos y pueden acceder a servicios de salud sexual y reproductiva; sin embargo, en la práctica, tiende a prevalecer la decisión adulta^14^. Esto se explica además por la centralidad que posee el criterio adulto en nuestra cultura, lo que merma las posibilidades de ejercer los derechos en la adolescencia desde un rol más protagónico, que les reconoce como actores sociales^11^^,^^13^^,^^31^. En este sentido, la edad es una categoría social^4^^,^^29^ que va más allá de derechos y deberes respecto de las leyes vigentes, ya que forma parte de representaciones sociales de la edad y generación, de ahí que las ideas preconcebidas de profesionales de salud inciden en la atención que brindan, y, por ende, en las posibilidades de decidir autónomamente. Si bien esta perspectiva podría incidir en la atención que brindan a la población general de adolescentes, a la luz de los resultados de este estudio, las ideas preconcebidas que existen respecto a formar parte de programas de protección y reinserción potencian esta perspectiva paternalista moderna y jurídica, al generalizar *a priori* respecto de su trayectoria de vida, asumiendo que requieren una atención diferenciada, que no tendrán adherencia al proceso y/o que por ello requieren presencia adulta para decidir respecto de su sexualidad, por ejemplo, al momento de elegir un método anticonceptivo o, en términos generales, para favorecer la adherencia y/o cumplir con requerimientos institucionales. 

Respecto de la autonomía corporal, entendida en los Objetivos de Desarrollo Sostenible (ODS) como las posibilidades que tienen mujeres de 15 a 49 años de recibir atención en salud, utilizar métodos anticonceptivos y negarse a mantener relaciones sexuales con una pareja, según indican informes recientes de Naciones Unidas en relación con el estado de la población mundial, la gran mayoría de las mujeres no tiene derecho a decidir sobre sus cuerpos en materia de salud sexual y reproductiva^6^^,^^20^^,^^32^^,^^33^. Los países que presentan informes al organismo internacional sobre este indicador señalan que solo el 56% de la población femenina a nivel mundial puede tomar decisiones informadas sobre sexo y reproducción^20^. En este sentido, se considera relevante trascender la mirada individual de la autonomía entendida como autosuficiencia, para comprenderla desde lo colectivo y relacional al reconocer la importancia de las relaciones e interacciones sociales para favorecer condiciones materiales y simbólicas para su ejercicio situado^13^. La autonomía progresiva en sexualidad está explícita en la Convención sobre los Derechos del Niño e incluye el derecho a recibir información con base científica sobre anticoncepción, ITS, VIH, aborto sin riesgos, en un espacio de atención confidencial^34^. 

Considerando lo anterior, quienes se desempeñan en el ámbito de la salud sexual y reproductiva cumplen un rol clave para reconocer su autonomía corporal/progresiva de las adolescencias como sujetos de derecho desde una mirada integral, dejando de lado la labor paternalista de las instituciones al decidir conforme los mandatos de género que la asociaban eminentemente a la maternidad^35^. Para mayor contexto y caracterizar la población adolescente en Chile respecto de dos de las dimensiones que se consideran para determinar niveles de autonomía corporal -atención en salud y uso de método anticonceptivo- es importante mencionar que, del total de la población un 13% de adolescentes entre 10 y 19 años se encuentra inscrito en el Fondo Nacional de Salud (FONASA)^36^^)^ y, del total de la población que ha iniciado un método anticonceptivo hormonal y/o utiliza preservativos, un 13% son adolescentes^37^. Si bien en este sentido, existen condiciones para el ejercicio de la autonomía corporal en términos de la cobertura y disponibilidad de anticonceptivos, las ideas preconcebidas de quien los provee, como también la desinformación que existe en temas de educación sexual integral^6^ inciden en el contexto en el que se desarrolla esta atención y, por ende, en las posibilidades que tienen para ejercer esta autonomía. 

Respecto de la perspectiva centrada en la autonomía, cabe destacar que los énfasis que se realizan con relación a resguardar la confidencialidad, favorecer un espacio seguro, considerar las especificidades que poseen y entregar herramientas para la toma de decisiones dan cuenta del reconocimiento de la salud sexual y reproductiva como derecho humano. Por lo tanto, la existencia de ideas paternalistas y adultocéntricas, que conviven con este discurso centrado en los derechos, podría reforzar estigmas al incluir juicios como antecedentes en la anamnesis al asumir, por ejemplo, que la sexualidad de este grupo de adolescentes estaría vinculada exclusivamente al trauma y durante la atención poner el foco en los factores de riesgo por sobre factores protectores y recursos personales. En la experiencia de este estudio, lo anterior se explicaría por el desconocimiento de quienes se desempeñan en salud respecto de la labor que desarrollan los programas de protección y justicia, como también respecto de las características de la población adolescente que forma parte de su cobertura^8^. 

Desde esta mirada centrada en el ejercicio de derechos, es importante visibilizar las particularidades que presentan las personas, con el objetivo de favorecer una mejor adherencia, manejo, acogida, atención y seguimiento en el sistema de salud; lo que en ningún caso pretende fortalecer prejuicios y/o estereotipos, sino que, por el contrario, visibilizar contextos, exclusiones, desventajas estructurales y determinantes sociales, para favorecer una mejor comprensión de la realidad que experimentan. Para incorporar esta perspectiva de análisis y visibilizar cómo los factores de exclusión se potencian y determinan privilegios y desventajas en las sociedades, Kimberlé Williams Crenshaw, abogada y académica, en 1989 propuso el concepto de interseccionalidad para visibilizar este sistema de opresión y analizar los diferentes componentes de la discriminación que, al superponerse, generan mayores obstáculos para el acceso y ejercicio de derechos^38^^,^^39^. 

En el ámbito de la salud, en documentos técnicos del Ministerio de Salud de Chile, la interseccionalidad se encuentra integrada de manera transversal al considerarse como una mirada que permite visibilizar especificidades que presentan las personas^40^^,^^41^ y específicamente a la población adolescente^42^, lo que está declarado de igual forma en orientaciones técnicas en el ámbito de la protección y reinserción^43^. En este sentido, se considera que el concepto de interseccionalidad no solo es un aporte desde el punto de vista teórico para enriquecer el análisis, sino que está declarado en los lineamientos institucionales que se encuentran vigentes a nivel intersectorial y que rigen la atención en salud. Al respecto, hablamos de una población específica de adolescentes, quienes más allá del género, experimentan otros aspectos de su identidad que se constituyen en factor de exclusión: edad, clase, etnia, cultura, nivel socioeconómico, condición migratoria, orientación sexual, entre otras, que acentúan sus brechas de acceso a diferentes espacios, entre ellos, la atención en salud. 

Esta consideración aplica a la población adolescente en general, no solo a grupos específicos, lo que refuerza la premisa de que la atención no debiera diferenciarse si incorpora los énfasis establecidos a nivel ministerial^40^^,^^41^^,^^42^. En este contexto, lo que sí se sugiere ajustar son los aspectos administrativos vinculados al acceso, particularmente, en el caso de adolescentes que han vivido vulneraciones de derechos. No se trata de establecer una atención diferenciada en el box, sino de implementar medidas de gestión que faciliten su acceso y permanencia en el sistema de salud^8^.

## Conclusiones

A partir de las experiencias expuestas, se identificaron perspectivas contrapuestas que conviven en la atención que realizan: una paternalista y una centrada en la autonomía y los derechos. Así mismo, se observó la disposición *a priori* a desarrollar una atención diferenciada, en tanto existen ideas preconcebidas respecto a adolescentes de programas de protección y justicia, que instan a asumir que existe una experiencia de la sexualidad centrada exclusivamente en el trauma y el riesgo. Estas preconcepciones, al actuar sobre dos de las dimensiones de autonomía corporal, inciden en las posibilidades que tendrán las adolescentes de ejercer sus derechos sexuales y derechos reproductivos, constituyendo una brecha. Esto se sustenta en ideas preconcebidas que generalizan sus vivencias de vulneración de derechos y conductas al margen de la ley, que es importante revisar con mayor detenimiento a nivel de equipos para evitar su reproducción y favorecer condiciones materiales para el ejercicio de derechos.

Se considera que las políticas públicas vigentes en Chile, que pretenden la priorización de esta población de adolescentes, a través de esfuerzos que fomenten la labor intersectorial entre salud, protección y reinserción, constituyen un avance en cuanto a la necesaria disminución de brechas respecto del acceso. Sin embargo, es importante que con ello no se refuercen ideas preconcebidas respecto de este grupo, sino que, por el contrario, se potencie la formación de profesionales a nivel de intersector. A partir de este estudio, se reafirma que la atención en salud sexual y reproductiva debe enfocarse en la entrega de herramientas para favorecer la toma de decisiones autónomas, involucrando activamente a las adolescencias en este proceso, en tanto las experiencias de vida que llevaron a pertenecer a estos programas no deben determinar su autonomía de decidir en sexualidad. 

En tanto la edad constituye una categoría social que se intersecciona con el género, el nivel socioeconómico y otras experiencias que se determinan como factores de exclusión, al considerar la autonomía como relacional le asigna al mundo adulto y a la institucionalidad la responsabilidad de contribuir a que existan condiciones que favorezcan el ejercicio de derechos protagónico, en el que se les reconozca su agencia como actores sociales, cuestionando la visión que aún sitúa a las adolescencias como sujetos de protección, pese a normativas nacionales e internacionales que las reconocen como sujetos de derechos. Una mirada interseccional en salud favorece no solo la comprensión de las determinaciones sociales que las personas encarnan y que les afecta su calidad de vida, sino que además facilita el desprendimiento de nociones preconcebidas basadas en estereotipos y favorece una atención libre de juicios, lo que en el caso de las adolescencias es vital para el ejercicio de derechos sexuales y reproductivos.
